# A Collection of Bioactive Nitrogen-Containing Molecules from the Marine Sponge *Acanthostrongylophora ingens*

**DOI:** 10.3390/md17080472

**Published:** 2019-08-15

**Authors:** Germana Esposito, Linh H. Mai, Arlette Longeon, Alfonso Mangoni, Emilie Durieu, Laurent Meijer, Rob Van Soest, Valeria Costantino, Marie-Lise Bourguet-Kondracki

**Affiliations:** 1 The Blue Chemistry Lab, Department of Pharmacy, University of Naples Federico II, 80138 Napoli, Italy; 2 Laboratoire Molécules de Communication et Adaptation des Micro-organismes, UMR 7245 CNRS, Muséum National d’Histoire Naturelle, 57 rue Cuvier (C.P. 54), 75005 Paris, France; 3 ManRos Therapeutics, Perharidy Peninsula, 29680 Roscoff, France; 4 Naturalis Biodiversity Center, P.O. Box 9517, 2300 RA Leiden, The Netherlands

**Keywords:** *Acanthostrongylophora ingens*, marine sponge, halicyclamine derivative, diketopiperazine, alkaloid, bipiperidine scaffold, kinase inhibitor, antimicrobial activity, inhibitor of amyloid β-42

## Abstract

Thirteen nitrogen-containing molecules (**1a**/**1b** and **2**–**12**) were isolated from the Indonesian sponge *Acanthostrongylophora ingens*, highlighting the richness of this organism as a source of alkaloids. Their structures were elucidated using one- and two-dimensional NMR spectroscopy and HR-ESI-MS, while the stereochemistry of the diketopiperazines was established using Marfey’s method. All compounds were screened in our standard bioactivity assays, including antibacterial, antikinases, and amyloid β-42 assays. The most interesting bioactivity result was obtained with the known acanthocyclamine A (**3**), which revealed for the first time a specific *Escherichia coli* antimicrobial activity and an inhibitory effect on amyloid β-42 production induced by aftin-5 and no cytotoxicity at the dose of 26 µM. These results highlight the potentiality of a bipiperidine scaffold as a promising skeleton for preventing or reducing the production of amyloid β-42, a key player in the initiation of Alzheimer’s disease.

## 1. Introduction

Over the last thirty years, research programs focusing on the chemical study of the metagenomic content of marine sponges have led to a plethora of novel molecules, often showing innovative skeletons, and being used as lead compounds in the search for novel therapeutic approaches [1,2,3,4].

In the framework of our research program, named BlueGenics (https://cordis.europa.eu/result/rcn/193365_en.html), devoting to the sustainable exploitation of bioactive marine compounds, the chemical composition of the dichloromethane extract of the marine sponge *Acanthostrongylophora ingens*, collected off the coast of South Sulawesi, Indonesia, was analyzed, and chloromethylhalicyclamine B (**5**), a novel selective CK1δ/ε kinase inhibitor with an IC_50_ value of 6 µM, was described [5]. An in-depth re-examination of the organic extracts of *A. ingens* revealed, in addition to halicyclamine B (**4**) and chloromethylhalicyclamine B (**5**), small amounts of tetradehydrohalicyclamine B (**1b**) and its epimer, a new dehydrohalicyclamine derivative named *epi*-tetradehydrohalicyclamine B (**1a**) as well as its chloromethyl derivative (**2**), acanthocyclamine A (**3**), and seven diketopiperazines (DKPs) (**6**–**12**). Tetradehydrohalicyclamine B (**1b**) was recently reported from the same Indonesian *A. ingens* by a Japanese group [6]. They investigated the proteasome inhibitor activity of tetradehydrohalicyclamine B (**1b**) and halicyclamine B (**4**), showing that both have proteasome inhibitor activity at micromolar concentration, **4** being more potent than **1b**, suggesting their possible role as anticancer lead compounds [7].

Our screening program includes antibacterial, antikinases, and amyloid β-42 assays. The obtained data are reported here, together with structural elucidation of the new alkaloid *epi*-tetradehydrohalicyclamine B (**1a**) found in the sponge extract.

## 2. Results

**Figure 1:**
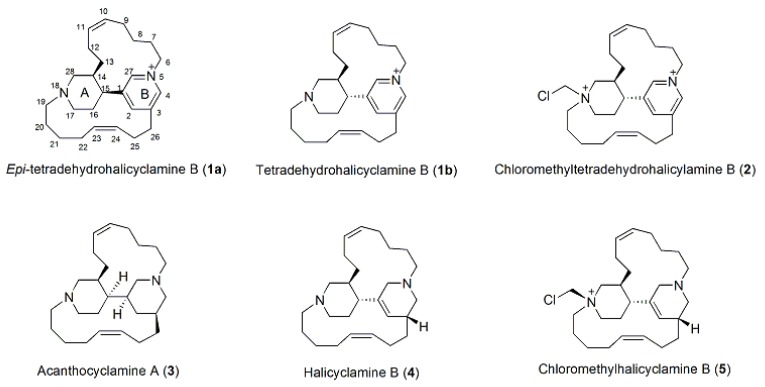
Structures of compounds **1**–**5**.

A sample of *A. ingens*, collected off South Sulawesi, Indonesia was extracted using our standard procedure as reported in the previous paper [5]. The CH_2_Cl_2_ and BuOH extracts were subsequently flash chromatographed on silica gel, yielding fractions (A1–A6) and (C1–C18), respectively, that were further purified as described in the experimental part and gave a mixture of *epi*-tetradehydrohalicyclamine B (**1a**) and tetradehydrohalicyclamine B (**1b**), the chloromethylderivative of **1b** (**2**), acanthocyclamine A (**3**), halicyclamine B (**4**), chloromethylhalicyclamine B (**5**) (Figure 1), and seven diketopiperazines (**6**–**12**). The known compounds **3**, **4**, and **5** were easily identified by comparison of their NMR and mass-spectrometry data with those reported in the literature [5,8].

### 2.1. Epi-Tetradehydrohalicyclamine B (***1a***) and Tetradehydrohalicyclamine B (***1b***)

**Figure 2:**
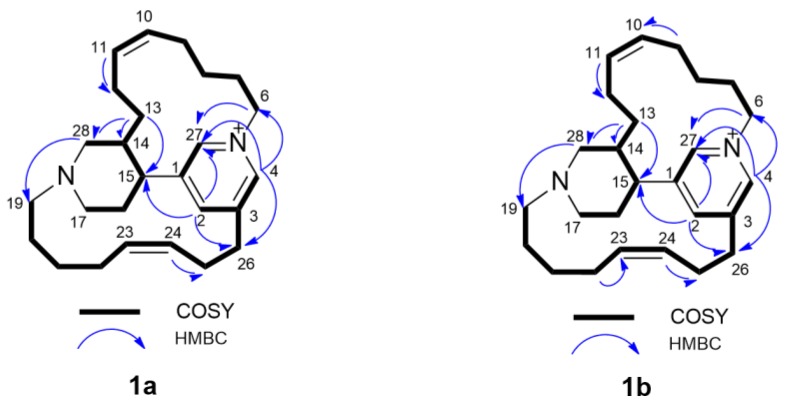
Most significant correlations provided by the COSY and HMBC NMR spectra of *epi*-tetradehydrohalicyclamine B (**1a**) and tetradehydrohalicyclamine B (**1b**).

Compounds **1a** and **1b** could not be obtained in pure form, but only as mixtures enriched in either **1a** or **1b**. These mixtures, allowing a clear distinction of the ^1^H and ^13^C signals of either stereoisomer, could be successfully used for structure elucidation. Compound **1a** showed the same molecular formula C_26_H_39_N_2_^+^ as the recently reported [6] tetradehydrohalicyclamine B (**1b**), accounting for 9 degrees of unsaturation, as deduced by the [M]^+^ peak at *m/z* 379.3132 and the doubly charged [M + H]^2+^ peak at *m/z* 190.1596 in the HR-ESIMS. Analysis of the one- and two-dimensional (COSY, TOCSY, ROESY, HSQC, and HMBC) NMR spectra of **1a** allowed the full assignment of all the ^1^H and ^13^C signals of both compounds **1a** and **1b**, and showed for **1a** the same planar structure as **1b** (Figure 2). Comparison of ^1^H NMR chemical shifts and coupling constants of compound **1a** (Table 1) with those of compound **1b** (Table S1) highlighted some remarkable differences, clearly implying a stereochemical difference between **1a** and **1b**. This could be linked not only to a different configuration at C-14 or at C-15, but also to atropisomerism (conformers separated by a high-energy barrier) of the same configurational stereoisomer, which is reasonable in the strained tetracyclic structure of **1a**/**1b**, and has been observed in other natural products [9]. The most notable feature of the ^1^H NMR spectrum of **1a** was the presence of two very shielded signals at δ_H_ 0.20 (H-8a) and δ_H_ 0.02 (H-13a) (in contrast, the same protons resonate at δ_H_ 1.01 and 1.16 respectively, in compound **1b**). The abnormally shielded chemical shift of H-8a and H-13a can be explained if these protons lie in the shielding zone of the pyridine ring and/or of the double bonds in the predominant conformation(s) of **1a**.

To discriminate between configurational stereoisomerism and atropisomerism, the conformational behavior of **1b** was studied. A conformational search was performed by molecular dynamics (MD), because MD-based methods are more suitable than Monte Carlo methods for flexible polycyclic molecules [10]. A series of 10-ns MD simulations was performed using the CFF91 force field, and geometries were extracted every 50 ps, resulting in 200 conformations from each MD simulations. Because we were looking for conformational changes much slower than the ns timescale at room temperature, simulation was performed at increasing temperatures, from 600 K to 3000 K. Overall, 108 conformers were obtained for tetradehydrohalicyclamine B (**1b**), considering only low-energy conformers in a range of 5 kcal/mol, and the sets of conformers obtained from the different simulations were very similar. These results suggested that no slow conformational equilibrium occurs for **1b**, thus excluding the hypothesis of atropisomerism.

Therefore, it was assumed that **1a** is a diastereomer of **1b**, i.e., the stereoisomer with *cis* substituents at C-14 and C-15. Unfortunately, the relative configuration of C-14 and C-15 could not be confirmed on the basis of the coupling constants of H-14 and H-15. In fact, even though C-14 and C-15 are part of a six-membered piperidine ring, the ring is in equilibrium between a chair and a twist-boat conformation (see below); in addition, the almost coincident chemical shifts of H-14 and H-16b, and of H-28a and H-28b produced a non-first-order spin system. Therefore, structure **1a** was validated using quantum-mechanical prediction of ^1^H and ^13^C chemical shift and ^1^H-^1^H coupling constants (see Table S2). The conformational space of compound **1a** was explored using again an MD-based conformational search, performed at 300 K. The search generated 42 conformers within 4 kcal/mol from the lowest-energy conformer. The geometry of each conformer was optimized quantum mechanically at the B3LYP/6-31G(d) level of theory and the continuum-solvent (PCM) model for MeOH, and ^1^H-^1^H coupling constants were calculated for each conformer at the B3LYP/6-31G(d,p) level of theory, according to the suggestion by Bally and Rablen [11]. Boltzmann-averaged coupling constants were then calculated, and compared with the respective experimental values. An excellent agreement was found between calculated and experimental coupling constants (Table 2), which strongly supported both the relative stereochemistry of structure **1a** and the quality of the conformational search.

**Figure 3:**
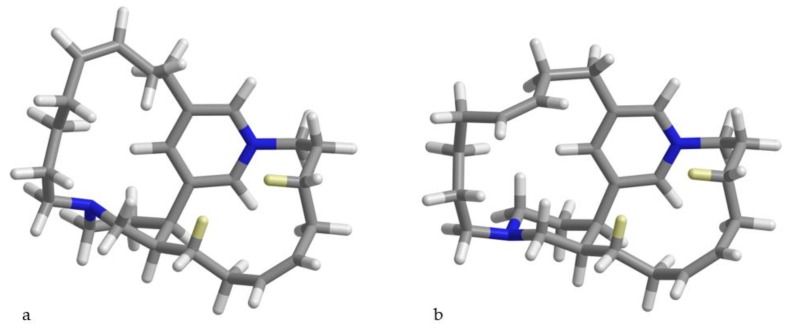
Representative conformations of compound **1a** with (**a**) the piperidine ring in twist-boat conformation and (**b**) the piperidine ring in the chair conformation. The shielded protons H-8a and H-13a are marked in yellow.

Examination of the conformers produced by the conformational search revealed an unexpected conformational rigidity of the C-6/C-13 carbon chain (containing the shielded protons H-8a and H-13a), which showed the same conformation in all the 16 lowest-energy conformers, accounting for over 96% of population. In this conformation, H-8a and H-13a (marked in yellow in Figure 3) are located in the shielding cone of the pyridine ring and in the shielding cone of the double bond at position 10, thus accounting for their unusually shielded chemical shift. In contrast, the C-19/C-26 carbon chain showed a much higher degree of flexibility. Finally, the piperidine ring showed an equilibrium between chair and twist-boat conformations, with the twist-boat largely dominating and accounting for over 78% of population (Figure 3). This conformational change involves pyramidal inversion at the nitrogen atom, and the minor chair conformation, with the lone pair towards the outside of the molecule, is important in the reaction with dichloromethane leading to compound **2** (see below).

Because compound **1a** could not be obtained in pure form, it was not possible to measure its optical rotation nor its electronic circular dichroism (ECD) spectrum. Therefore, its absolute configuration could not be established experimentally, but was assumed to be (14*R*,15*R*) by analogy with absolute configuration of acanthocyclamine A (**3**) [8].

### 2.2. Chloromethyltetradehydrohalicyclamine B (***2***)

Compound **2** was isolated as a colorless solid. Analysis of the HR-ESIMS showed a doubly charged [M]^2+^ ion peak at *m/z* 214.1473, with an M+2 isotope peak whose intensity (35% compared to M) suggested the presence of a chlorine atom in the molecule. The molecular formula was deduced as C_27_H_41_ClN_2_^2+^, indicating nine degrees of unsaturation in the molecule no single-charge ion was observed in the MS spectrum, suggesting the presence of two quaternary ammonium nitrogen atoms in the molecule. Detailed examination of NMR spectra revealed that compound **2** had the same atom connectivity as in compounds **1a** and **1b** (Figure 1), except for an additional methylene signal (δ_H_ 5.38, δ_C_ 69.9). This was indicative of a chloromethyl group linked to N-18, as observed in chloromethylhalicyclamine B (**5**) [5]. Consequently, this product resulted is an artifact produced during the extraction process, but its structure was not studied in depth because it did not reveal any activity in our panel of assays.

### 2.3. Diketopiperazines ***6**–**12***

**Figure 4:**
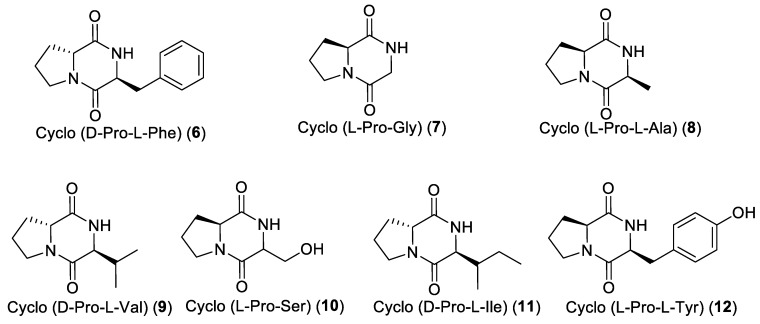
Structures of diketopiperazines **6**–**12** isolated from *Acanthostrongylophora ingens*.

In addition, seven diketopiperazines were isolated (Figure 4). Their planar structures were easily identified by their spectroscopic data in comparison with those found in the literature, while the absolute configurations of their stereogenic carbons were determined using Marfey’s analyses. Therefore, after hydrolysis and derivatization with the L-enantiomer of Marfey’s reagent, the obtained derivatives were analysed by HR-ESIMS-HPLC. On the basis of the retention times of their respective Marfey’s derivatives, the absolute configuration of the stereogenic carbons of the seven diketopiperazines were deduced as depicted in Figure 4. As for the cyclo (Pro-Ser) (**10**), only the proline residue was determined as L-Pro.

### 2.4. Evaluation of Biological Activities in the Antibacterial, Antikinases, and Amyloid β-42 Assays

All the isolated compounds were tested against the bacteria *Staphylococcus aureus* and *Escherichia coli* as well as against eight different protein kinases relevant to cell proliferation, cancer, diabetes, and neurodegenerative disorders (CDK1, CDK2, CDK5, CDK9, CK1, CLK1, DYRK1A, and GSK3) and in both amyloid β-42 assays (amyloid β-42 induction assay and inhibition of amyloid β-42 production induced by aftin-5 assay). The main significant results are presented in Table 3. Only acanthocyclamine A (**3**) and halicyclamine B (**4**) showed a selective antimicrobial activity against *E. coli* and *S. aureus*, respectively (diameter inhibition of 12 and 10 mm at 100 µg/disk, respectively). Furthermore, while chloromethylhalicyclamine B (**5**) showed a selective inhibitory activity against the protein kinase CK1δ/ε with an IC_50_ value of 6 µM [5], the diketopiperazine cyclo(d-Pro-l-Phe) (**6**) displayed a selective kinase inhibitory activity against CDK2/cyclin A with an IC_50_ value of 1 µM. The amyloid β-42 assays were performed preliminary on both CH_2_Cl_2_ and BuOH extracts, showing an inhibition of the amyloid β-42 production induced by aftin-5, without reducing survival of the N2a-APP695 cell line. Their activity was confirmed in dose-response (0.1, 1.0 and 10 µg/mL). Therefore, **1**–**12** were tested in both amyloid β-42 assays at the dose of 10 µg/mL. Among the compounds tested, acanthocyclamine A (**3**), showed an inhibition of amyloid β-42 production induced by aftin-5 at 26 µM, without cytotoxicity at this dose.

## 3. Discussion and Conclusions

Alkaloids are pharmacologically well characterized and are used in therapy, ranging from chemotherapeutics to analgesics.

This chemical study of the marine sponge *A. ingens* allowed the isolation of 13 alkaloids, of which, one, **1a** is the epimer of the tetradehydrohalicyclamine B (**1b**), just recently published. Compounds **1**–**12** were analyzed for their biological activity using our standard panel assays that include antibacterial, antikinases, and amyloid β-42 assays. Acanthocyclamine A (**3**) showed a selective antimicrobial activity against *E. coli* and an inhibition of the amyloid β-42 production induced by aftin-5 at 26 µM, without cytotoxicity at this dose. These results highlight the potentiality of a bipiperidine scaffold as a promising skeleton to develop products able to prevent or reduce the production of amyloid β-42, a key player in the initiation of Alzheimer’s disease. A previous study revealed that chloromethylhalicyclamine B (**5**) appeared to be a selective CK1δ/ε inhibitor at low micromolar concentrations, while halicyclamine B (**4**) was inactive. Docking studies showed that chloromethylhalicyclamine B (**5**) can efficiently interact with the ATP-binding site of CK1δ in spite of its globular structure, very different from the planar structure of known inhibitors of CK1δ [5]. Because no CK1δ inhibitory activity was observed for the chloromethyltetradehydrohalicyclamine B (**2**), the presence of a tetrahydropyridine appears to be essential for the inhibitory activity towards CK1δ.

Moreover, the diketopiperazine cyclo (d-Pro-l-Phe) (**6**) revealed a selective antikinase activity against CDK2/cyclin A with an IC_50_ value of 1 µM. In comparison with the inactive diketopiperazine cyclo (L-Pro-L-Tyr) (**12**), we can hypothesize that hydroxylation of the phenyl group leads to a loss of CDK2/cyclin A kinase inhibitory activity.

The marine sponge *A. ingens* is showed to be a rich source of a number of bioactive alkaloids, some of them having an unusual skeleton, and sometimes being halogenated.

This study completes the previous works on *A. ingens* and points out the growing interest in studying this sponge family with unique and diverse chemical structures. These data highlight the potentiality of these molecules as lead compounds.

## 4. Materials and Methods

### 4.1. General Experimental Procedures

Mass spectra were recorded on an API Q-STAR PULSAR I (Applied Biosystem, Concord, ON, Canada). NMR spectra were obtained on either a Bruker Avance 400 or 600 spectrometer (Bruker, Wissenbourg, France) using standard pulse sequences. The acquisition of HMBC spectra were optimized for either 7 or 8.3 Hz. Other NMR spectra were recorded on Varian Unity Inova spectrometers at 700 MHz (Agilent Technology, Cernusco sul Naviglio, Italy); chemical shifts were referenced to the residual solvent signal (CD_3_OD: δ_H_ 3.31, δ_C_ 49.00). Flash chromatography was carried out on Buchi C-615 pump system (Rungis, France). Analytical and semi-preparative reversed-phase (Gemini C6-phenyl, Phenomenex, Le Pecq, France) columns were performed with an Alliance HPLC apparatus (model 2695, Waters, Saint-Quentin-en-Yvelines, France), equipped with a photodiode array detector (model 2998, Waters), an evaporative light-scattering detector (model Sedex 80, Sedere, Alfortville, France), and the software Empower (Waters). Chromatography columns (CC) were performed using silica gel (200~400 mesh; Merck, Darmstadt, Germany) and Sephadex LH-20 (Amersham Pharmacia, Uppsala, Sweden).

The Marfey’s experiments were performed using a Thermo LTQ Orbitrap XL mass spectrometer coupled to a Thermo Ultimate 3000 RS system (Thermo Fisher Scientific Spa, Rodano, Italy), which included solvent reservoir, in-line degasser, ternary pump, column thermostat, and refrigerated autosampler. LC–MS data were recorded and analyzed using the software Thermo Xcalibur 2.07 (Thermo Fisher Scientific Spa). The samples (5 μL) were applied on to an analytical reversed-phase column (Phenomenex Kinetex C18, 100 × 2.1 mm, particle size 5 μm), which was eluted at 200 μL/min. The elution procedure consisted of an isocratic profile of acetonitrile–water (5:95, *v*/*v*) for 3 min, followed by a linear gradient from 5% to 60% ACN/H_2_O over 20 min, a linear gradient from 60% to 90% ACN/H_2_O over 1 min, and an isocratic profile over 5 min.

### 4.2. Sponge Material

Specimens of *Acanthostrongylophora ingens* (Thiele, 1900) (class Demospongiae, order Haplosclerida, family Petrosiidae) were collected off the South Sulawesi, Indonesia and Makassar (Ujong Pandang), Spermonde Archipelago, north-west Lankai Island, reef slope at 14 m depth, 5.019° S 119.063° E, 29 April 1998, coll. B.W. Hoeksema, and were identified by one of the authors (R.V.S.) at the University of Amsterdam, The Netherlands, where a voucher specimen was deposited under the registration code ZMA Por. 14471.

### 4.3. Isolation and Purification

Sponge specimens (500 g) were immediately immersed in MeOH after collection. The MeOH solution was evaporated and the aqueous residue was extracted and partitioned successively with CH_2_Cl_2_ (1L), EtOAc (1L), and BuOH (1L) to obtain the corresponding extracts: Extract A (CH_2_Cl_2_, 2.5 g), extract B (EtOAc, 1.6 g), and extract C (BuOH, 8.5 g).

The CH_2_Cl_2_ extract was subsequently flash chromatographed on silica gel using a gradient elution system from 100% CH_2_Cl_2_ to 100% MeOH, obtaining six fractions (A1–A6).

Fraction A3 (313 mg) was further fractionated into 14 sub-fractions on silica gel column chromatography eluted with the CH_2_Cl_2_–acetone gradient system. Sub-fraction A3-7 (22 mg) was subjected to C6-phenyl semi-preparative reversed-phase HPLC using, as eluent, the gradient ACN/H_2_O/HCOOH from 5/95/0.1 to 50/50/0.1 for 35 min (flow rate: 3 mL/min, wavelength: 201 nm) and yielded the diketopiperazine cyclo-(D-Pro-L-Ile) (**11**, 4 mg). From the sub-fraction A3-8 (17 mg), the three diketopiperazines cyclo-(D-Pro-L-Val) (**9**, 5 mg), cyclo-(L-Pro-L-Tyr) (**12**, 3 mg) and cyclo(D-Pro-L-Phe) (6, 4 mg) were purified after C6-phenyl semi-preparative reversed-phase HPLC using the gradient ACN/H_2_O/HCOOH from 5/95/0.1 to 28/72/0.1 for 27 min (flow rate: 3 mL/min, wavelength: 201 nm).

Fraction A5 (250 mg) was applied on a Sephadex LH-20 column using MeOH as eluent and gave eleven sub-fractions. Sub-fraction A5-3 (8 mg) was subjected to C6-phenyl semi-preparative reversed-phase HPLC using the gradient ACN/H_2_O/HCOOH from 5/95/0.1 to 40/60/0.1 for 11 min (flow rate: 3 mL/min, wavelength: 201 nm) to yield 3 mg of a mixture of **1a** and **1b** with a ratio of 65:35 by ^1^H NMR integration.

Fraction A6 (150 mg) was applied on a Sephadex LH-20 column and eluting with MeOH to give nine sub-fractions. Sub-fraction A6-2 was purified by C6-phenyl analytical reversed-phase HPLC using the gradient ACN/H_2_O/HCOOH from 5/95/0.1 to 13/87/0.1 for 25 min (flow rate: 1 mL/min, wavelength: 201 nm) to yield chloromethyltetradehydrohalicyclamine B (**2**, 1.2 mg). Using a gradient system ACN/H_2_O/HCOOH (5/95/0.1 to 20/80/0.1 for 25 min, flow rate 1 mL/min, wavelength 254 nm), sub-fraction A6-9 was purified by C6-phenyl analytical reversed-phase HPLC yielding chloromethylhalicyclamine B (**5**, 2.5 mg) and halicyclamine B (**4**, 1.8 mg).

An aliquot of the BuOH extract (2 g) was subsequently flash chromatographed on a silica gel column using the elution gradient system from 100% CH_2_Cl_2_ to 100% MeOH, to yield eighteen fractions (C1-C18). Fraction C6 was subjected to C6-phenyl semi-preparative reversed-phase HPLC using ACN/H_2_O/HCOOH from 5/95/0.1 to 35/65/0.1 as elution gradient for 25 min (flow rate: 3 mL/min, wavelength: 201 nm) and yielded cyclo-(L-Pro-Gly) (**7**, 2 mg), cyclo-(L-Pro-L-Ala) (**8**, 2.5 mg), and cyclo-(L-Pro-Ser) (**10**, 2.7 mg).

Fraction C13 was applied on a Sephadex LH-20 column using MeOH as eluent and yielded three sub-fractions. Sub-fraction C13-2 was subjected to C6-phenyl semi-preparative reversed-phase HPLC using the gradient ACN/H_2_O/HCOOH from 5/95/0.1 to 25/75/0.1 for 28 min (flow rate: 3 mL/min, wavelength: 201 nm) to yield acanthocyclamine A (**3**, 5 mg).

*Epi*-tetradehydrohalicyclamine B (**1a**): ^1^H and ^13^C NMR data, see Table 1, (+)- HR-ESIMS *m/z* 379.3132 [M]^+^ (calcd for C_26_H_39_N_2_^+^, 379.3107).

### 4.4. Marfey’s Analysis

Marfey’s analysis was used to determine the configuration of the amino acids of DKPs cyclo(Pro-Phe), cyclo(Pro-Gly), cyclo(Pro-Ala), cyclo(Pro-Val), cyclo(Pro-Ser), cyclo(Pro-Ile), and cyclo(Pro-Tyr). The compounds were subjected to hydrolysis and derivatization with the L-enantiomer of Marfey’s reagent (FDAA, or 1-fluoro-2,4-dinitrophenyl-5-alanine amide) [12]. Only 10 μg of compounds were degraded, and the obtained derivatives were analyzed by HR-ESIMS-HPLC.

The samples were treated with 6 N HCl and heated in a flame-sealed glass tube at 180 °C for 2 h. The residual HCl fumes were removed in vacuo. The hydrolysate of the diketopiperazines were dissolved in triethylamine/ACN (2:3) (100 µL), and these solutions were then treated with 1-fluoro-2,4-dinitrophenyl-5-l-alaninamide (L-FDAA) in ACN/acetone (1:2) (100 μL). The vials were heated at 50 °C for 1 h. The mixture was dried and re-suspended in ACN/H_2_O (5:95) (500 μL) for subsequent LC-MS analysis. Authentic L-Phe, L-Pro, L-Tyr, L-Val, L-Ile, L-Ala standards were treated with L-FDAA and D-FDAA.

In spite of the low amounts used, the extracted-ion chromatograms of the diketopiperazines at *m*/*z* 418.1357 (FDAA-Phe), 368.1201 (FDAA-Pro), 434.1306 (FDAA-Tyr), 370.1357 (FDAA-Val), 384.1514 (FDAA-Ile), and 342.1044 (FDAA-Ala) were almost devoid of noise.

**Figure 5:**
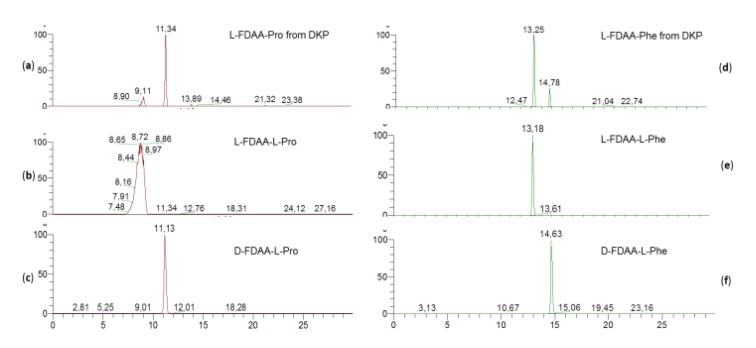
HR-ESIMS-HPLC analysis of Marfey’s derivatives from DKP cyclo(Pro-Phe) (**6**): Extracted-ion chromatograms at *m/z* 368.1201 of L-1-fluoro-2,4-dinitrophenyl-5-alanine amide (FDAA)-Pro from DKP (**a**); of authentic L-FDAA-L-Pro (**b**); and of authentic D-FDAA-L-Pro (**c**); extracted-ion chromatograms at *m/z* 418.1357 of L-FDAA-Phe from DKP (**d**); of authentic L-FDAA-L-Phe (**e**); and of authentic D-FDAA-L-Phe (**f**).

In DKP cyclo(Pro-Phe) (**6**), the phenylalanine residue was found to have L configuration while the proline residue was found to have D configuration on the basis of the retention times of their respective Marfey’s derivatives (Figure 5).

In DKP cyclo(Pro-Gly) (**7**), L configuration was found for proline residue (Figure S1).

In DKP cyclo(Pro-Ala) (**8**), both alanine and proline residues were found to have L configuration (Figure S2).

In DKP cyclo(Pro-Val) (**9**), the valine residue was found to have L configuration while the proline residue was found to have D configuration (Figure S3).

In DKP cyclo(Pro-Ser) (**10**), L configuration was found for proline residue while it was not possible to establish the configuration for the serine residue, probably because of the low amount of diketopiperazine (Figure S4).

In DKP cyclo(Pro-Ile) (**11**), the isoleucine residue was found to have L configuration while the proline residue was found to have the D configuration (Figure S5).

In DKP cyclo(Pro-Tyr) (**12**), the tyrosine and the proline residues were found to have the L configuration (Figure S6).

### 4.5. Quantum Mechanical Prediction of ^1^H-^1^H Coupling Constants of epi-Tetradehydrohalicyclamine B (***1a***)

Initial conformational search was performed using molecular dynamics simulations at different temperatures, performed in vacuo in the CFF91 force field using the Insight II/Discover package (BIOVIA: San Diego, CA, USA). The resulting conformers (42 conformers within 4 kcal/mol from the lowest-energy conformer) were used as starting structure for quantum mechanical calculations with the Gaussian 09 program [13]. Geometries were optimized at the B3LYP/6-31G(d) level of theory and the continuum-solvent (PCM) model for MeOH, and ^1^H-^1^H coupling constants were calculated for each conformer at the B3LYP/6-31G(d,p) level of theory according to the suggestion by Bally and Rablen [11], i.e., considering only the Fermi contact contribution to *J* and scaling the calculated value by 0.9117. The averaged coupling constants shown in Table 2 were obtained using populations calculated from the CFF91 energies, and including in the calculations only the conformers populated by more than 1%. It is interesting to note that using quantum mechanical calculations to evaluate energies and populations of conformers produced a remarkably worse fit with the experimental values. The inaccuracy of relative energies of conformers is a known problem of DFT calculations [10], which in this particular study could not be avoided even when a higher level of theory (e.g., B3LYP-D3/TZVP) was used.

### 4.6. Antimicrobial Assays

Assays were performed using the disk diffusion assay method. Pure compounds (100 µg) were solubilized in DMSO and deposited on a 6 mm paper disk and put on agar plated seeded with reference strains *Staphylococcus aureus* (ATCC 6538) and *Escherichia coli* (ATCC 8739). Antimicrobial activity was determined by measuring the diameter of the inhibition zone after 24 h of incubation at 37 °C. Cefotaxime (30 µg) and amoxicillin (25 µg) were used as positive controls against *S. aureus* and *E. coli*, giving 30 and 21 mm of inhibition zones, respectively.

### 4.7. Antikinases Assays

Evaluation of the protein kinase activity was performed in vitro as previously described [14]. Briefly, the buffers used in the experiments were prepared as follows: Homogenization buffer: 60 mM β-glycerophosphate, 15 mM *p*-nitrophenylphosphate, 25 mM Mops (pH 7.2), 15 mM EGTA, 15 mM MgCl_2_, 1 mM dithiothreitol, 1 mM sodium vanadate, 1mM NaF, 1 mM phenylphosphate, 10 µg leupeptin/mL, 10 µg aprotinin/mL, 10 µg soybean trypsin inhibitor/mL, and 100 µg benzamidine; buffer A: 10 mM MgCl_2_, 1mM EGTA, 1 mM dithiothreitol, 25 mM Tris-HCl pH 7.5, 50 µg heparin.mL**^−^**^1^; buffer C: Homogenization buffer but 5 mM EGTA, no NaF and no protease inhibitors. Kinase activities were assayed in duplicates in buffer A or C at 30 °C, at a final ATP concentration of 15 µM. The order of mixing the reagents was: Buffers, substrate, enzyme, and inhibitor. Isolated compounds were tested against a panel of eight kinases; namely, dual-specificity tyrosine-(Y)-phosphorylation regulated kinase 1A (DYRK1A), cyclin-dependent kinase 5 (CDK5/p25), glycogen synthase kinase-3 (GSK-3 α/β), CDC-like kinase 1 (CLK-1), casein kinase 1 (CK1δ/ε), cyclin-dependent kinase 1 (CDK1/cyclin B), cyclin-dependent kinase 2 (CDK2/cyclin A), and cyclin-dependent kinase 9 (CDK9/cyclin T).

### 4.8. Amyloid β42 Induction Assay

This assay, described in detail in reference [15] allows the detection of molecules able to induce the production of extracellular amyloid β-42 peptide.

N2a cells stably transfected with human APP695 were maintained in Dulbecco’s modified Eagle’s media (DMEM/optiMEM, Gibco, InVitrogen, St. Aubin, France), supplemented with 5% fetal bovine serum (Gibco, InVitrogen, St. Aubin, France), 1% penicillin-streptomycin solution (Sigma Aldrich, Saint- Quentin Fallavier, France), and G418 (0.1 mg/mL) in a humidified atmosphere at 37 °C with 5% CO_2_. N2a-APP695 cells were plated at a density of approximately 10,000 cells per well in 96-well plates in modified media (DMEM/optiMEM) with 0.5% FBS. After 18 h incubation, the conditioned media were replaced by new media containing compounds at the final concentrations of 0.1, 1.0, 10 μg/mL. After 18 h incubation, the cultured media were harvested for amyloid β-42 determination by ELISA assay.

Amyloid β-42 levels were measured in a double antibody sandwich ELISA using a combination of monoclonal antibody (mAb) 6E10 (SIG-39320, Covance, Eurogentec, Seraing, Belgium) and biotinylated polyclonal amyloid β-42 antibody (provided by Dr. P.D. Mehta, Institute for Basic Research in Developmental Disabilities, Staten Island, NY, USA). Briefly, wells of microtiter plates (Maxisorp, Nunc, ThermoFisher Scientific, Illkirch, France) were coated 100 μL mAb 6E10 diluted in carbonate-bicarbonate buffer (buffer (0.015 M Na_2_CO_3_ + 0.035 M NaHCO_3_) pH 9.6) at a 1.5 μg/mL final concentration, and plates were incubated overnight at 4 °C. The plates were then washed with PBST (PBS containing 0.05% Tween-20) and blocked for 1 h with 1% BSA in PBST to avoid non-specific binding. Following a washing step, 100 μL of cell supernatant was added and incubated for 2 h at room temperature (RT) on a shaking device. Plates were then washed with PBST and 100 μL of biotinylated antibodies (diluted to 1 μL/mL in PBST containing 0.5% BSA) were added and incubation was carried out for 75 min at RT under constant shaking. After a washing step, streptavidin-Poly-HRP (horseradish peroxidase) conjugate (Pierce, ThermoFisher Scientific, Illkirch, France), diluted in PBS + 1% BSA, was added and incubation was carried out for 45 min at RT under continuous shaking. After washing, 100 μL of OPD (*o*-Phenylenediamine dihydrochloride, Pierce, ThermoFisher Scientific, Illkirch, France) in pH 5.0 citrate buffer (0.049M citric acid monohydrate + 0.1M Na_2_HPO_4_·2H_2_O + 1 mL H_2_O_2_ 30%/L) were added as a substrate and after 15 min incubation at room temperature, the reaction was stopped by addition of 100 μL 1 N sulfuric acid. Optical density (OD) was measured at 490 nm using a plate reader (BioTek Instruments, El 800, Gen 5 software, Winooski, VT, USA).

Amyloidβ–42 quantification was calculated using standard curves, which were prepared with synthetic Aβ-42 HFIP treated (JPT Peptide Technologies, Berlin, Germany) and Aβ-42 specific polyclonal antibody. Curve fitting was performed using a 4 parameters sigmoid equation (SigmaPlot, Systat, Sigma). Results are expressed as fold change ± s.d. All experiments were performed in triplicate.

### 4.9. Inhibition of Amyloid β-42 Production Induced by Aftin-5 Assay

This assay allows the detection of molecules able to inhibit the production of extracellular Amyloid β peptides induced by a pre-treatment with 100 μM of aftin-5. Aftin-5 is available from Adipogen International, San Diego, CA, USA.

N2a cells stably transfected with human APP695 were maintained in Dulbecco’s modified Eagle’s media (DMEM/optiMEM), supplemented with 5% fetal bovine serum, 1% penicillin-streptomycin solution (Sigma), and G418 (0.1 mg/mL) in a humidified atmosphere at 37 °C with 5% CO_2_. N2a-APP695 cells were plated at a density of approximately 10,000 cells per well in 96-well plates in modified media (DMEM/optiMEM) with 0.5% FBS. After 18 h incubation, the conditioned media were replaced by new media containing compounds at the final concentrations of 0.1, 1.0, 10 μg/mL. After 1 h incubation, aftin-5 was added (100 μM 1% DMSO final). After 18 h incubation, the cultured media were harvested for amyloid β-42 determination by ELISA assay.

### 4.10. Cytotoxic Assay: Effects on N2a-APP695 Viability (MTS Survival Assay)

This assay allows evaluating the survival rate of cultured mammalian cells exposed to extracts or pure compounds. It allows the detection of cell death-inducing molecules.

N2a cells stably transfected with human APP695 were maintained in Dulbecco’s modified Eagle’s medium (DMEM/optiMEM), supplemented with 5% fetal bovine serum, 1% penicillin-streptomycin solution, and G418 (Sigma, St. Louis, MO, USA) (0.1 mg/mL) in a humidified atmosphere at 37 °C with 5% CO_2_.

N2a-APP695 cells were plated at a density of approximately 10,000 cells per well on 96-well plates in Dulbecco’s modified Eagle’s medium (DMEM/optiMEM), supplemented with 0.5% fetal bovine serum. After 24 h incubation, the conditioned media were replaced by new media containing compounds at the final concentrations of 0.1, 1.0, 10 µg/mL. Viability of cells was measured by MTS-formazan reduction using CellTiter 96 Aqueous One Solution Cell Proliferation Assay (Promega, Madison, WI, USA) at 18 h post treatment. Incubation was pursued for 1.5 h (37 °C, 5% CO_2_, and 95% humidity). Optical density (OD) was measured at 490 and 630 nm using a microELISA reader (BioTek Instruments, Winooski, VT, USA.).

## Figures and Tables

**Table 1:** NMR data of *epi*-tetradehydrohalicyclamine B (**1a**) (700 MHz, CD_3_OD).

Pos.		δ_H_ [mult., *J* (Hz)]	δ_C_ (mult.)	COSY	HMBC	NOESY
1			146.2 (C)			
2		9.44 s	149.4 (CH)	2, 4, 26a	4, 15, 26, 27	16a, 21a, 21b, 25a, 25b, 26a, 28
3			145.7 (C)			
4		8.73 s	141.5 (CH)	2, 6b, 26a, 27	2, 3, 6, 26, 27	6b, 7b, 26a, 26b
5						
6	a	4.64 ddd (13.4, 13.4, 4.1)	60.0 (CH_2_)	6b, 7a, 7b, 27	4, 7, 27	7a, 9b, 27
	b	4.84 ddd (13.4, 5.6, 1.5)		6a, 7a, 7b	4, 7, 8, 27	4, 7a, 7b
7	a	2.02 br. dd (14.6, 13.3)	30.2 (CH_2_)	6a, 6b, 8a, 8b		6a, 6b, 7b
	b	2.23 m		6a, 6b, 7a, 8a, 8b		4, 6b, 7a, 8a, 8b
8	a	0.20 ddddd (14.6, 13.3, 13.1, 4.0, 2.9)	26.4 (CH_2_)	7a, 7b, 8b, 9a, 9b		7b, 8b, 9b, 12a
	b	1.54 ddddd (14.6, 14.0, 4.1, 4.0, 4.0)		7a, 7b, 8a, 9b		8a, 10
9	a	1.62 dddd (13.2, 13.2, 5.5, 4.0)	25.5 (CH_2_)	8a, 9b, 10	8, 10, 11	8a, 9b, 10
	b	2.58 dddd (14.0, 13.0, 9.0, 4.1)		8a, 9a, 10	10, 11	8a, 13a
10		5.48 m	130.1(CH)		9	8b, 9b
11		5.45 m	130.9 (CH)	12	12	12, 13a, 13b
12	a	1.68 tt (13.0, 4.3)	25.0 (CH_2_)	11, 13a, 13b	13	13a
	b	2.58 dddd (14.0, 14.0, 8.5, 4.0)				
13	a	0.02 dddd (14.3, 14.3, 11.4, 4.0)	35.0 (CH_2_)	12, 13b, 14	12, 14	8a, 9b, 13b, 28
	b	1.31 dddd (14.3, 14.3, 4.0, 3.0)		12, 13a, 14	15, 28	11, 12, 13a, 14
14		2.30 m	37.1 (CH)	13a, 13b, 15, 16a	28	12, 13b, 15, 16a, 17b, 19a
15		3.56 ddd (9.8, 5.2, 1.4)	36.4 (CH)	14, 16a	1, 2, 14, 16, 17, 27, 28	16a, 16b, 17a, 27
16	a	2.09 dddd (14.4, 10.3, 7.6, 1.4)	30.5 (CH_2_)		1, 13, 14, 15, 17	2, 15, 17b
	b	2.32 m		15, 16a, 17a, 17b	1, 15	15, 16a, 17a
17	a	2.55 ddd (11.6, 10.3, 5.2)	46.8 (CH_2_)	16a, 16b, 17b	13	
	b	2.86 ddd (11.6, 7.6, 2.8)		16a, 16b, 17a	13, 15, 16, 19, 28	
18						
19	a	2.55 m	57.5 (CH_2_)	20b, 21a	17, 21, 28	
	b	2.62 m		20a, 21b	16, 17, 28	
20	a	1.58 m	26.6 (CH_2_)	19b, 21b	20	19b, 21b
	b	1.68 m		19a, 21a, 21b, 22b	19	
21	a	1.55 m	28.3 (CH_2_)	21b	19, 20, 22	2, 22a
	b	1.74 m		19b, 21a, 22a, 22b	19, 20, 22	2, 21a, 22b
22	a	2.04 dddd (13.5, 10.2, 7.8, 5.5)	27.7 (CH_2_)	21a, 21b, 22b, 23	21	2
	b	2.20 m		21a, 21b, 22a, 23, 25b	21	21a, 21b, 22a, 23, 25b
23		5.75 ddd (10.1, 8.2, 8.2)	132.2 (CH)	22a, 22b, 24	22, 25	21a, 22a, 22b, 24
24		5.48 m	130.4 (CH)	23, 25a, 25b	22, 25	23, 25b, 26a, 26b
25	a	2.49 dddd (15.2, 10.0, 8.2, 4.8)	28.8 (CH_2_)	24, 26a, 26b	3, 23, 24, 26	
	b	2.56 dddd (15.2, 8.8, 8.2, 7.0)		24, 26a, 26b	3, 23, 24, 26	
26	a	2.85 ddd (14.6, 8.8, 4.8)	33.6 (CH_2_)	25a, 25b	2, 3, 4, 24, 25	
	b	3.08 ddd (14.6, 8.2, 8.2)		25a, 25b, 26a	2, 3, 4, 24, 25	4, 25b, 26a
27		9.04 s	144.2 (CH)	1, 2, 4, 6a	2, 4, 6, 15	6a, 8a, 9b, 12a, 15
28		2.35 m	54.8 (CH_2_)		13, 14, 17, 19	2, 13a, 13b, 16a, 17a, 20a

**Table 2:** Experimental and calculated ^1^H-^1^H coupling constants of *epi*-tetradehydrohalicyclamine B (**1a**).

Coupled Protons	Calculated ^3^*J*_H-H_ (Hz)	Experimental ^3^*J*_H-H_ (Hz)
H-6a/H-7a	13.6	13.4
H-6a/H-7b	3.9	4.1
H-6b/H-7a	5.8	5.6
H-6b/H-7b	1.6	1.6
H-7a/H-8a	2.7	2.9
H-7a/H-8b	4.0	4.1
H-7b/H-8a	13.2	13.1
H-7b/H-8b	3.0	4.0
H-8a/H-9a	13.1	13.3
H-8a/H-9b	3.3	4.0
H-8b/H-9a	4.3	4.0
H-8b/H-9b	14.2	14.0
H-9a/H-10	6.0	5.5
H-9b/H-10	10.3	9.0
H-11/H-12b	10.6	8.5
H-12a/H-13a	13.0	14.3
H-12a/H-13b	4.1	4.0
H-12b/H-13a	3.4	4.0
H-12b/H-13b	13.9	14.0
H-13a/H-14	11.6	11.4
H-13b/H-14	2.6	3.0
H-14/H-15	5.0	5.2
H-15/H-16a	1.3	1.4
H-15/H-16b	9.3	9.8
H-16a/H-17a	9.5	10.3
H-16a/H-17b	7.2	7.6
H-16b/H-17a	5.6	5.2
H-16b/H-17b	3.0	2.8

**Table 3:** Significant results of the biological activity evaluation of **1**–**6**.

Compound	*Antimicrobial* *Assays ^a^*		*Protein kinase* *Assays ^b^*		*Amyloid* *β-42* *Assays ^c^*		*Cytotoxic* *Assay ^d^*
	*S. aureus*	*E. coli*	*CK1δ*/*ε*	*CDK2*/*cyclin A*	*Induction*	*Inhibition*	N2a-APP695
1a/1b	ND	ND	NA	NA	NA	NA	NA
2	ND	ND	NA	NA	NA	NA	NA
3	ND	12 mm	NA	NA	NA	26 µM	NA
4	10 mm	ND	NA	NA	NA	NA	NA
5	ND	ND	6 µM	NA	NA	NA	NA
6	ND	ND	NA	1 µM	NA	NA	NA

^a^ Expressed as inhibition diameter (mm) at 100 µg/disk; ^b^ Expressed as inhibitory activity (IC_50_ in µM); ^c^ Effect on amyloid β-42 level; ND: No activity detected at 100 µg/disk; NA: Not active at the dose tested (10 µg/mL).

## Supplementary Materials

The following are available online at https://www.mdpi.com/1660-3397/17/8/472/s1, Table S1. NMR data of tetradehydrohalicyclamine B (**1b**) (700 MHz, CD_3_OD), Table S2. Cartesian coordinates, relative energies, and populations of the nine lowest-energy conformers of *epi*-tetradehydrohalicyclamine B (**1a**). Figure S1. HR-ESI-MS-HPLC analysis of Marfey’s derivatives from DKP cyclo(Pro-Gly) (**7**), Figure S2. HR-ESI-MS-HPLC analysis of Marfey’s derivatives from DKP cyclo(Pro-Ala) (**8**), Figure S3. HR-ESI-MS-HPLC analysis of Marfey’s derivatives from DKP cyclo(Pro-Val) (**9**), Figure S4. HR-ESIMS-HPLC analysis of Marfey’s derivatives from DKP cyclo(Pro-Ser) (**10**), Figure S5. HR-ESI-MS-HPLC analysis of Marfey’s derivatives from DKP cyclo(Pro-Ile) (**11**), Figure S6. HR-ESI-MS-HPLC analysis of Marfey’s derivatives from DKP cyclo(Pro-Tyr) (**12**).
