# Supplementary material for: A Collection of Bioactive Nitrogen-Containing Molecules from the Marine Sponge Acanthostrongylophora ingens
Source: Mar Drugs. 2019 Aug 15;17(8):472. doi: 10.3390/md17080472 (PMC6723059; doi:10.3390/md17080472)
Supplement: Supplementary file 1 [file marinedrugs-17-00472-s001.pdf]

Supplementary Information

Table S1. NMR data of tetradehydrohalicyclamine B (**1b**) (700 MHz, CD_3OD).

Table S2. The experimental and theoretically predicted values of the indirect spin-spin coupling constants [Hz] for *epi*-tetradehydrohalicyclamine B (**1a**). NMR parameters were calculated at the mPW1PW91/6-31G(d,p) level of theory.

Figure S1. HR-ESIMS-HPLC analysis of Marfey's derivatives from DKP cyclo(Pro-Gly) **(7)**

Figure S2. HR-ESIMS-HPLC analysis of Marfey's derivatives from DKP cyclo(Pro-Ala) **(8)**

Figure S3. HR-ESIMS-HPLC analysis of Marfey's derivatives from DKP cyclo(Pro-Val) **(9)**

Figure S4. HR-ESIMS-HPLC analysis of Marfey's derivatives from DKP cyclo(Pro-Ser) **(10)**

Figure S5. HR-ESIMS-HPLC analysis of Marfey's derivatives from DKP cyclo(Pro-Ile) **(11)**

FigureS6. HR-ESIMS-HPLC analysis of Marfey's derivatives from DKP cyclo(Pro-Tyr) **(12)**

Pos.		$δ_H$ [mult., *J* (Hz)]	$δ_C$ [mult.]	COSY	HMBC	NOESY
1			147.3 (C)			
2		8.43 s	144.6 (CH)	3, 4, 26	15, 26, 27	14, 16b, 19b, 24, 25, 26, 27
3			144.5 (C)			
4		8.81 s	143.9 (CH)	2, 6b, 26	3, 6, 26, 27	6b, 7a, 26
5						
6	a	4.60 ddd(12.4,12.1,3.9)	62.1 (CH_2)	7a, 7b, 27	3, 7, 27	7a, 7b, 8a, 8b, 9b, 27
	b	4.77 dt(13.5,4.2)		4, 7a, 7b	3, 7, 8, 27	4, 7a, 7b
7	a	2.09 m	29.6 (CH_2)	6a, 6b, 8b		4, 6a, 6b, 8a, 8b
	b	2.16 m		6a, 6b, 8a		6a, 6b, 8a, 8b, 27
8	a	1.01 m	26.7 (CH_2)			
	b	1.66 m		7a, 8a, 9a	6, 9	6a, 7a, 7b, 8a, 10, 27
9	a	1.28 dddd (14.5, 10.3, 9.6, 3.9)	26.9 (CH_2)	8b, 9b	10	
	b	2.06 m		8b, 9a, 10	8, 10	
10		5.30 m	130.6 (CH)			
11		5.31 m	129.4 (CH)		12	
12	a	1.44 m	26.2 (CH_2)	11, 12b, 13	10, 13, 14	11, 12b, 14
	b	2.05 m		12a		11, 12a, 15, 27
13	a	1.16 dddd(13.9, 9.8, 8.8, 3.0)	33. 9(CH_2)	12a	12, 14, 15	
	b	1.22 m				
14		1.72 m	38.7 (CH)	13, 15, 17a, 28a		2, 12a, 13, 16b, 28b
15		2.97 ddd(9.0,8.0,6.8)	42.4 (CH)	14, 16a, 16b	1, 13, 14, 16, 27	12b, 13, 16a, 27, 28a
16	a	2.01 m	23.9 (CH_2)	15, 16b, 17a, 17b		15, 16b, 17a
	b	2.38 m		15, 16a, 17a, 17b		2, 16a, 19b
17	a	2.99 m	50.5 (CH_2)	16a	15	15, 16a, 17b, 28a
	b	3.25 m		16a, 16b	15, 28	16b, 17a, 19a
18						
19	a	2.69 m	49.7 (CH_2)	19b, 20		19b, 20
	b	3.10 m		19a, 20		2, 20
20		1.75 m	23.0 (CH_2)	19a, 19b	21	19a, 28b
21		1.59 m	26.0 (CH_2)	22a, 22b	19, 22, 23	22a, 22b
22	a	2.12 m	25.3 (CH_2)	21, 22b, 23		20, 21, 22b, 23
	b	2.54 m		21, 22a, 23	21, 23	
23		5.50 m	133.3 (CH)	22a, 22b, 24	22	20, 21, 22a, 22b, 23
24		5.22 m	130.3 (CH)	22a, 23, 25	25	2, 22b, 25b, 26
25		3.00 m	25.2 (CH_2)	24, 25b, 26		2, 25a
26	a	3.10 dddd (18.5, 11.2, 4.1)	30.9 (CH_2)	2, 25	2, 24, 25	2, 4, 24, 25
	b	3.18dt (17.0, 4.4)				
27		8.86 s	142.7 (CH)	2, 6a, 26	4, 6, 15	6a, 8b, 9a, 9b, 15
28	a	2.49 m	53.6 (CH_2)	14, 28b	14, 19	13, 15, 17a, 28b
	b	3.04 m		14, 28a		13, 28a

Table S1. NMR data of tetradehydrohalicyclamine B (**1b**) (700 MHz, CD_3OD).

Formatted: Not Highlight
Formatted: Not Highlight
Formatted: Not Highlight

	Predicted ^3JH-H	Experimental ^3JH-H
6a-7a	11.7	13.4
6a-7b	3.7	4.1
6b-7a	5.4	5.6
6b-7b	1.6	1.5
7a-8a	2.7	2.9
7a-8b	3.9	4.1
7b-8a	11.5	13.1
7b-8b	2.9	4.0
8a-9a	11.4	13.3
8a-9b	3.3	4.0
8b-9a	4.1	4.0
8b-9b	12.4	14.0
9a-10	6.3	5.5
9b-10	8.8	9.0
11-12b	9.0	8.5
12a-13a	11.4	14.3
12a-13b	4.0	4.0
12b-13a	3.4	4.0
12b-13b	12.2	14.0
13a-14	9.9	11.4
13b-14	2.8	3.0
14-15	5.2	5.2
15-16a	0.9	1.4
15-16b	10.3	9.8
16a-17a	9.7	10.3
16a-17b	7.8	7.6
16b-17a	5.4	5.2
16b-17b	0.6	2.8

Formatted: Not Highlight

Formatted: Not Highlight

Table S2. The experimental and theoretically predicted values of the indirect spin-spin coupling constants [Hz] for *epi*-tetradehydrohalicyclamine B (**1a**). NMR parameters were calculated at the mPW1PW91/6-31G(d,p) level of theory.

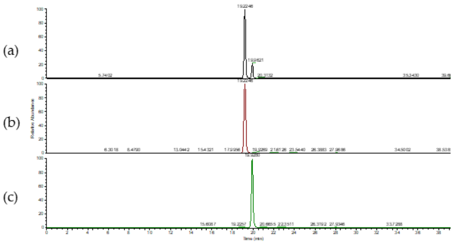

Figure S1. HR-ESI-MS-HPLC analysis of Marfey's derivatives from DKP cyclo(Pro-Gly) **(7)**
Extracted-ion chromatograms at *m*/*z* 368.1201 of L-1-fluoro-2-4-dinitrophenyl-5-alanine amide (FDAA)-Pro from DKP(**a**); of authentic L-FDAA-L-Pro (**b**); and of authentic D-FDAA-L-Pro (**c**). The proline residue was found to have L configuration on the basis of the retention times of its Marfey's derivative.

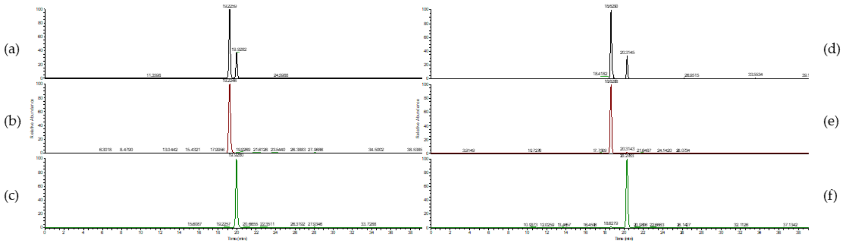

Figure S2. HR-ESI-MS-HPLC analysis of Marfey's derivatives from DKP cyclo(Pro-Ala) **(8)**
Extracted-ion chromatograms at *m*/*z* 368.1201 of L-1-fluoro-2-4-dinitrophenyl-5-alanine amide (FDAA)-Pro from DKP(**a**); of authentic L-FDAA-L-Pro (**b**); and of authentic D-FDAA-L-Pro (**c**); extracted-ion chromatograms at *m*/*z* 342.1044 of L-FDAA-Ala from DKP (**d**); of authentic L-FDAA-L-Ala (**e**); and of authentic D-FDAA-L-Ala (**f**). The alanine and proline residues were found to have L configuration.

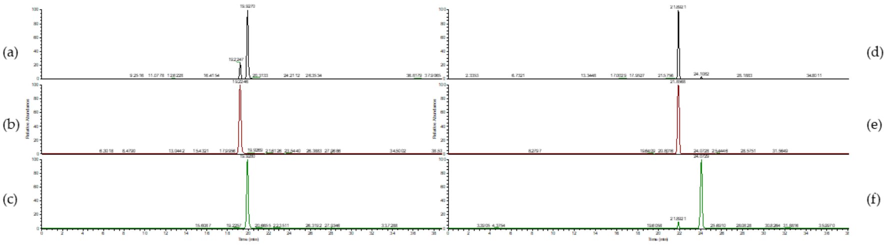

Figure S3. HR-ESI-MS-HPLC analysis of Marfey's derivatives from DKP cyclo(Pro-Val) **(9)**
Extracted-ion chromatograms at *m*/*z* 368.1201 of L-1-fluoro-2-4-dinitrophenyl-5-alanine amide (FDAA)-Pro from DKP(**a**); of authentic L-FDAA-L-Pro (**b**); and of authentic D-FDAA-L-Pro (**c**); extracted-ion chromatograms at *m*/*z* 370.1357 of L-FDAA-Val from DKP (**d**); of authentic L-FDAA-L-Val (**e**); and of authentic D-FDAA-L-Val (**f**). The valine residue was found to have L configuration while the proline residue was found to have D configuration.

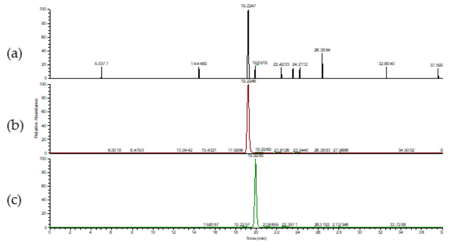

Figure S4. HR-ESI-MS-HPLC analysis of Marfey's derivatives from DKP cyclo(Pro-Ser) **(10)**
Extracted-ion chromatograms at *m*/*z* 368.1201 of L-1-fluoro-2-4-dinitrophenyl-5-alanine amide (FDAA)-Pro from DKP(**a**); of authentic L-FDAA-L-Pro (**b**); and of authentic D-FDAA-L-Pro (**c**). The proline residue was found to have the L configuration while for the serine residue was not possible to establish the configuration probably because of the low amount of DKP cyclo (Pro-Ser).

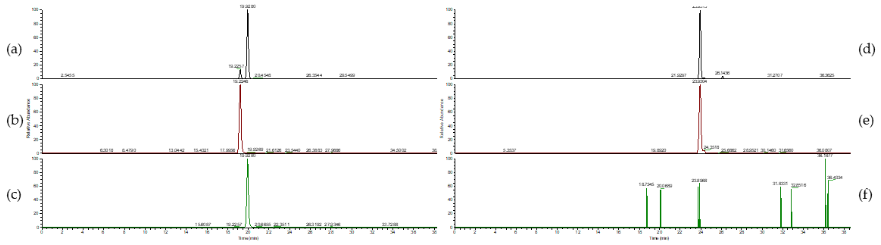

Figure S5. HR-ESI-MS-HPLC analysis of Marfey's derivatives from DKP cyclo(Pro-Ile) **(11)**
Extracted-ion chromatograms at *m*/*z* 368.1201 of L-1-fluoro-2-4-dinitrophenyl-5-alanine amide (FDAA)-Pro from DKP(**a**); of authentic L-FDAA-L-Pro (**b**); and of authentic D-FDAA-L-Pro (**c**); extracted-ion chromatograms at *m*/*z* 384.1514 of L-FDAA-Ile from DKP (**d**); of authentic L-FDAA-L-Ile (**e**); and of authentic D-FDAA-L-Ile (**f**). L configuration was found in isoleucine residue while D configuration was found in proline residue.

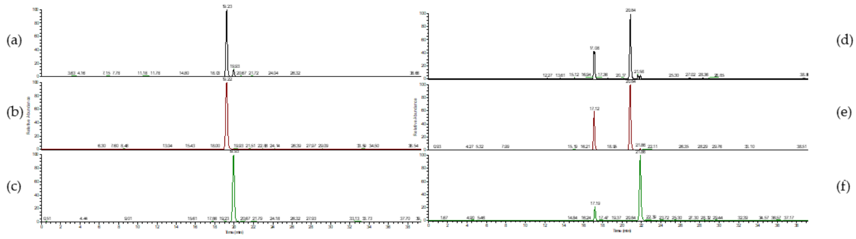

Figure S6. HR-ESI-MS-HPLC analysis of Marfey's derivatives from DKP cyclo(Pro-Tyr) **(12)**
Extracted-ion chromatograms at *m*/*z* 368.1201 of L-1-fluoro-2-4-dinitrophenyl-5-alanine amide (FDAA)-Pro from DKP(**a**); of authentic L-FDAA-L-Pro (**b**); and of authentic D-FDAA-L-Pro (**c**); extracted-ion chromatograms at *m*/*z* 434.1306 of L-FDAA-Tyr from DKP (**d**); of authentic L-FDAA-L-Tyr (**e**); and of authentic D-FDAA-L-Tyr (**f**). The tyrosine and the proline residues were found to have L configuration on the basis of the retention times of their respective Marfey's derivatives.
